# Synergy between oral PDE5 inhibitors and topically applied nitric oxide microparticles on the erectile response in a rat model of cavernous nerve injury

**DOI:** 10.1038/s41443-025-01147-x

**Published:** 2025-08-07

**Authors:** Moses T. Tar, Andrew Draganski, Kelvin P. Davies

**Affiliations:** 1 Department of Urology, Albert Einstein College of Medicine, Bronx, NY USA; 2 Zylo Therapeutics, Inc, Grenville, SC USA

**Keywords:** Drug delivery, Experimental models of disease

## Abstract

This study aimed to evaluate the effectiveness of combining a nitric oxide microparticle delivery system (NO-MP) with various FDA-approved PDE5 inhibitors (PDE5i) for improving erectile responses in a rat model of erectile dysfunction (ED) following cavernous nerve injury, similar to the effects of radical prostatectomy. Male Sprague-Dawley rats, 4–5 months old (weighing ~275 g) underwent bilateral cavernous nerve transection. One week post-surgery animals were administered PDE5i via oral gavage (sildenafil 0.05 mg/kg (*N* = 9), tadalafil 0.005 mg/kg (*N* = 8), vardenafil 0.01 mg/kg (*N* = 7), avanafil 0.1 mg/kg (*N* = 8) or untreated (*N* = 5) followed by topical application of 250 mg NO-MP to the penile dermis. Erectile responses were assessed by measuring intracorporeal pressure (ICP) and systemic blood pressure (BP) after the application of NO-MP. Compared to NO-MP alone, combination therapy with a PDE5i significantly (*P* < 0.05) reduced the time to initial erectile response from 63 ± 21.6 min to 8–23 min (vardenafil: 23 ± 2.3, avanafil: 11 ± 8.1, sildenafil: 19.9 ± 9.8, tadalafil: 18 ± 12.8), and increased the frequency of spontaneous erections from 1 ± 0.71 to 1.7–2.7 per hour (vardenafil: 2.1 ± 0.9, avanafil: 2.7 ± 1.1, sildenafil: 1.7 ± 0.6, tadalafil: 2.5 ± 0.8). No significant changes in maximal ICP/BP, duration of erectile response or baseline ICP/BP were observed. These results suggest that combining NO-MP with PDE5i may provide a promising approach for treating ED after radical prostatectomy.

## Introduction

Following surgery for radical prostatectomy (RP) used in the treatment of prostate cancer, men are at heightened risk for development of erectile dysfunction (ED) [1]. The *Prostate Cancer Outcomes Study* documented that approximately 60% of men experience self-reported ED 18 months after RP, and only 28% of men reported erections firm enough for intercourse at a five-year follow-up [1]. The main pathophysiological mechanism resulting in ED is injury to cavernous nerves (CN) [2, 3] which innervate erectile tissue. In an attempt to preserve nerve function, robotic surgical procedures aimed at nerve-sparing are now routinely used, although several recent reviews have found that even with these newer procedures erectile function outcomes after RP are not convincingly improved [4].

CN injury disrupts the release of nitric oxide (NO) from nerve endings that innervate erectile tissue. NO plays a crucial role in activating the molecular signaling pathways necessary for initiating an erection by increasing intracellular levels of cyclic guanosine monophosphate (cGMP) [5, 6]. In the absence of cGMP, phosphodiesterase type 5 inhibitors (PDE5i), which are the first-line FDA-approved oral therapies for ED [7, 8], become ineffective, as they work to maintain cGMP levels [9].

Prior studies have demonstrated that topically applied microparticles delivering NO (NO-MP) can generate an erectile response in animal models of both aging and RP [10, 11]. We propose that topical application of NO-MP to the penis by-passes the release of NO from CN endings and initiates the molecular signaling pathways that lead to an erectile response. Subsequent studies demonstrated synergy between orally administered sildenafil and NO-MP [12].

Four PDE5i are currently FDA-approved as oral treatments for ED: sildenafil (Viagra™) [13], avanafil (Stendra™) [14], vardenafil (Levitra™) [15], and tadalafil (Cialis™) [16]. While all these drugs’ target PDE5 and share similar safety profiles, they differ in their molecular structures, pharmacodynamics (such as onset time, selectivity, and duration of action), and pharmacokinetics (including absorption, bioavailability, metabolism, and half-life). Given these differences, the present study aimed to evaluate the effects of these four FDA-approved PDE5i on erectile function when administered orally in combination with topically applied NO-MP in an animal model of RP.

## Materials and Methods

### Animal Model

Animal use in this study was conducted in accordance with the Guide for the Care and Use of Laboratory Animals as outlined by the U.S. National Institutes of Health and was approved by the Animal Care and Use Committee at Albert Einstein College of Medicine. A total of 37 male Sprague-Dawley rats, aged 4–5 months and weighing approximately 275 g, were obtained from Charles River Laboratories (Boston, MA, USA). The rat model of RP involves bilateral transection of the CN (originally described in [17] and used in several of our published preclinical studies investigating the ability of MP to restore erectile function after CN injury [10, 11, 18]).

Briefly, the procedure involves a vertical low-abdominal midline incision to locate the major pelvic ganglion (MPG), situated on the dorsolateral lobes of the prostate [17]. The MPG receives inputs from the hypogastric and pelvic nerves, with the CN being one of its outputs. CN transection is performed by cutting the nerve 2–5 mm distal to the MPG under dissecting microscope visualization [17]. One week following surgery, the combined effects of an oral dose of a PDE5i and topical application of NO-MP on erectile function were assessed by measuring the intracorporal pressure to systemic blood pressure (ICP/BP) ratio, as previously described [19].

### Test Reagents

The NO-MP was produced by Zylo Therapeutic, Inc. (Greenville, SC, USA) by a procedure outlined in previous publications [12, 20]. Sildenafil, avanafil, tadalafil and vardenafil were obtained from Sigma-Aldrich Corp. (St. Louis, MO, USA).

### Experimental Procedure

The effect of topical application of 250 mg of NO-MP on erectile response in CN-transected rats was evaluated in combination with different PDE5i. Previous studies have demonstrated a significant synergistic effect when 250 mg of NO-MP was applied topically alongside an oral dose of 0.05 mg/kg sildenafil, which is roughly equivalent to a clinical dose of 2.5 mg for an 80 kg patient [21]. This dose is 10 times lower than the smallest commercially available sildenafil tablet (25 mg). Based on these findings, the current experiments utilized an oral dose of PDE5i that was 10 times less than the lowest commercially available dose, equivalent to patient doses of 0.25 mg tadalafil, 0.5 mg vardenafil, or 5 mg avanafil.

Prior to anesthesia, animals were administered PDE5i in 0.2 ml of aqueous solution via oral gavage (sildenafil 0.05 mg/kg (*N* = 9), tadalafil 0.005 mg/kg (*N* = 8), vardenafil 0.01 mg/kg (*N* = 7), avanafil 0.1 mg/kg (*N* = 8) or untreated (*N* = 5). Anesthesia was induced through an intraperitoneal injection of sodium pentobarbital (35 mg/kg), and a cannula was inserted into the carotid artery for continuous monitoring of BP. A perineal incision was made, and the ischiocavernosus muscle was removed to expose the corpus cavernosum. A 23-gauge needle was then inserted into the corpus cavernosum to measure ICP. Both ICP and BP were continuously monitored for approximately 2 h. Following a 30-minute baseline measurement of ICP and BP NO-MP was topically applied using a spatula along the dermis of the penile shaft (as depicted in supplementary Fig. 1). The following parameters were recorded: time to the initial erectile response, duration of the erectile response, maximal and average ICP/BP ratios, and the number of spontaneous erections per hour (in the context of these experiments “spontaneous erections” are defined as an increase in ICP/BP over baseline that occurs without direct physical stimulation).

### Analysis

Animals were randomly assigned to different treatment groups, and the investigator responsible for measuring ICP/BP was blinded to which PDE5i was being administered. No animals were excluded from analysis unless they developed pathology due to the surgical procedure. Data are presented as means with standard error of the mean (S.E.M). A modified t-test was employed to compare erectile responses—including the time to initial erection, duration of erection, maximal and average ICP/BP ratios, and the number of spontaneous erections per hour—between animals treated with NO-MP and NO-MP used in combination with a PDE5i. A *P*-value of <0.05 was considered statistically significant.

## Results

**Fig. 1:**
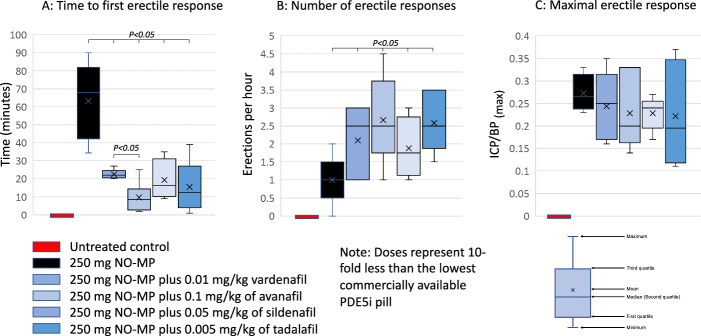
Rats were orally administered with PDE5i, 30 min prior to surgical prep for determination of erectile response by ICP/BP (doses equivalent to a human dose of 2.5 mg sildenafil (*N* = 9), 0.25 mg of tadalafil *N* = 8); 0.5 mg vardenafil (*N* = 7); or 5 mg of avanafil (*N* = 8)). Following topical application of 250 mg of NO-MP the **A** Average time to the first erectile response (Note: if no erectile response was seen, the course of the experiment ~90 min was used as value), **B** Average erectile responses per hour; **C** Average Maximal ICP/BP was determined. The error bars represent standard error.

In Fig. 1, we present studies investigating the erectile response in CN-transected rats, following treatment with a combination of topically applied NO-MP and four FDA-approved oral PDE5i: vardenafil, avanafil, sildenafil, and tadalafil. The rationale for the dosages used in these experiments is derived from our recently published study [12], in which a synergistic effect was observed between 250 mg of topically applied NO-MP and 0.05 mg/kg of orally administered sildenafil. This sildenafil dose is 10-fold lower than the lowest commercially available tablet (25 mg). Building on this finding, we examined the effect of combining 250 mg of topically applied NO-MP with doses of PDE5i that are 10-fold lower than the lowest available oral doses. Specifically, we used equivalent human doses of 0.25 mg tadalafil, 0.5 mg vardenafil, and 5 mg avanafil.

As shown in Fig. 1, untreated control animals did not exhibit spontaneous erections. However, topical application of 250 mg NO-MP resulted in the onset of spontaneous erections beginning at a mean of 63 ± 21.6 min after treatment and occurring at a rate of 1 ± 0.71 per hour. When oral PDE5i were combined with the topical NO-MP, both the time to the first erectile response (Fig. 1A) and the rate of spontaneous erections (Fig. 1B) were significantly altered. Compared to NO-MP alone, combination therapy with a PDE5i significantly (*P* < 0.05) reduced the time to initial erectile response to 8–23 min (vardenafil: 23 ± 2.3, avanafil: 11 ± 8.1, sildenafil: 19.9 ± 9.8, tadalafil: 18 ± 12.8), and increased the frequency of spontaneous erections to 1.7–2.7 per hour (vardenafil: 2.1 ± 0.9, avanafil: 2.7 ± 1.1, sildenafil: 1.7 ± 0.6, tadalafil: 2.5 ± 0.8). Combination treatment did not significantly affect the maximal ICP/BP ratio, with values remaining within a range of 0.2 to 0.26 (Fig. 1C). Similar to our previous study [12], NO-MP alone produced an erectile response lasting 6.5 ± 3.2 min, which was not significantly altered by co-administration with PDE5i. Baseline ICP remained unchanged following topical NO-MP in animals pre-treated with oral PDE5i.

## Discussion

The results of this study demonstrate that topical application of 250 mg NO-MP can induce spontaneous erections in untreated animals, with the first occurrence at a mean of 63 ± 21.6 min after treatment and occurring at a rate of 1 ± 0.71 per hour. To investigate a potential synergistic effect with NO-MP, we evaluated whether co-administration of a PDE5i at one-tenth the standard clinical dose could enhance erectile function. Even at this substantially reduced dose, combination therapy significantly improved both the time to the initial erectile and frequency of spontaneous erections. Compared to NO-MP alone, co-treatment with a PDE5i significantly (P < 0.05) decreased the time to initial erectile response from 63 ± 21.6 min to between 8 and 23 min (vardenafil: 23 ± 2.3, avanafil: 11 ± 8.1, sildenafil: 19.9 ± 9.8, tadalafil: 18 ± 12.8), and increased the frequency of spontaneous erections from 1 ± 0.71 to 1.7–2.7 per hour (vardenafil: 2.1 ± 0.9, avanafil: 2.7 ± 1.1, sildenafil: 1.7 ± 0.6, tadalafil: 2.5 ± 0.8). These results suggest a synergistic interaction between NO-MP and PDE5i that enhances functional outcomes even at lower doses. Such a strategy may allow effective treatment while minimizing the risk of dose-dependent adverse effects commonly associated with PDE5i monotherapy. Importantly, the combination did not significantly alter the maximal ICP/BP ratio, indicating that the improvements were primarily in the timing and frequency of erectile events rather than in the overall magnitude of the erectile response.

While the combination treatment improved erectile response time and frequency, it did not significantly alter the maximal ICP/BP ratio. In general, a visible erection in rat models is associated with an ICP/BP > 0.6, and although we observed a significant improvement in erectile responses over baseline, even the maximum erectile response observed was less that 0.35. In several other studies aimed at recovering erectile function after a period of CN injury, post-operative erectile responses (ICP/BP) are rarely normalized [22, 23]. This may indicate that while the combination therapy enhances the initiation and recurrence of spontaneous erections, it does not necessarily affect the peak intensity of the erectile response, which is likely influenced by other factors such as vascular tone, smooth muscle function, and nitric oxide availability. The lack of change in the ICP/BP ratio may also reflect the limits of this experimental model, where maximum ICP could be constrained by anatomical or physiological factors unrelated to the pharmacological treatments administered.

In addition, similar to prior studies [12], topical application of NO-MP alone produced an erectile response lasting 6.5 ± 3.2 min, which was not significantly altered by oral co-administration with PDE5i. For context, reflexive or apomorphine-induced erections in rats typically last less than 1 minute [24]. Baseline ICP also remained unchanged following a combination of topically applied NO-MP in animals pre-treated with oral PDE5i.

A limitation of our study is that we did not specifically assess whether avanafil and vardenafil induced spontaneous erections following administration. While these PDE5i share a similar mechanism of action with sildenafil, which has previously been shown, along with vardenafil, not to induce spontaneous erections in rat models of cavernous nerve injury within a two-hour observation window, this assumption was not directly tested for avanafil or vardenafil in our experiments.

The current study highlights the potential for combination therapies in the management of ED, particularly for patients who may benefit from a dual mechanism of action. Topical NO-MP may provide a localized and rapid onset of action, while oral PDE5i can sustain and amplify the erectile response over time. Further studies investigating the long-term effects and safety of this combination, as well as exploring additional doses and treatment regimens, are warranted to fully understand the potential clinical applications of this approach.

## Supplementary information

Supplemental Figure 1: NO-MP was topically applied using a spatula along the dermis of the penile shaft.

## Data Availability

Data analyzed in this paper is presented in Fig. 1.
