# Supplementary material for: Synergy between oral PDE5 inhibitors and topically applied nitric oxide microparticles on the erectile response in a rat model of cavernous nerve injury
Source: Int J Impot Res. 2025 Aug 7;38(6):486–9. doi: 10.1038/s41443-025-01147-x (PMC13249558; doi:10.1038/s41443-025-01147-x)
Supplement: Supplementary file 1 — Supplemental Figure 1: NO-MP was topically applied using a spatula along the dermis of the penile shaft. [file 41443_2025_1147_MOESM1_ESM.pdf]

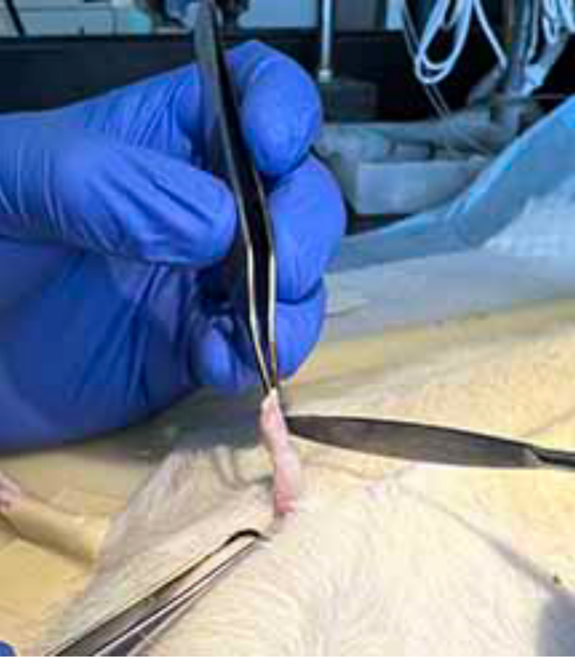

Supplemental Figure 1: NO-MP was topically applied using a spatula along the dermis of the penile shaft.
